# Ensuring HIV Data Availability, Transparency and Integrity in the MENA Region: Comment on "Improving the Quality and Quantity of HIV Data in the Middle East and North Africa: Key Challenges and Ways Forward"

**DOI:** 10.15171/ijhpm.2017.53

**Published:** 2017-05-22

**Authors:** Kayvon Modjarrad, Sten H. Vermund

**Affiliations:** ^1^ U.S. Military HIV Research Program, Walter Reed Army Institute of Research, Silver Spring, MD, USA.; ^2^ Henry M. Jackson Foundation, Bethesda, MD, USA.; ^3^ Yale School of Public Health, Yale University, New Haven, CT, USA.

**Keywords:** HIV, Surveillance, Middle East, North Africa, Data Accuracy, Health Policy

## Abstract

In this commentary, we elaborate on the main points that Karamouzian and colleagues have made about HIV data scarcity in Middle Eastern and North African (MENA) countries. Without accessible and reliable data, no epidemic can be managed effectively or efficiently. Clearly, increased investments are needed to bolster capabilities to capture and interpret HIV surveillance data. We believe that this enhanced capacity can be achieved, in part, by leveraging and repurposing existing data platforms, technologies and patient cohorts. An immediate modest investment that capitalizes on available infrastructure can generate data on the HIV burden and spread that can be persuasive for MENA policy-makers to intensify efforts to track and contain the growing HIV epidemic in this region. A focus on key populations will yield the most valuable data, including among men who have sex with men (MSM), transgender women and men, persons who inject drugs (PWIDs), female partners of high risk men and female sex workers.

The HIV/AIDS pandemic is now well into its fourth decade. Local conditions differ and regional epidemics have diverging trajectories. Since the antiretroviral and infrastructure investments spurred by the massive HIV programs began in 2003-2004—such as the US President’s Emergency Plan for AIDS Relief (PEPFAR) and the Global Fund to Fight AIDS, Tuberculosis and Malaria—HIV incidence and AIDS-related death rates have fallen significantly in some of the worst-affected areas of the world (ie*,* sub-Saharan Africa, Southeast Asia). In contrast, HIV has steadily risen in what were previously thought to be low-risk, low prevalence zones (ie, Eastern Europe, Central Asia, Middle East and North Africa [MENA]).^1^ Karamouzian et al, in their recent editorial, offer insights into one of the key reasons for this epidemiologic discrepancy: poor data availability and integrity.^2^ The authors identify some of the major challenges to capturing and validating HIV surveillance data in the MENA region; including the persistence of violent conflict, lack of political will, and stigmatization of the most-at-risk populations.

Although there are some common challenges that stretch across the region, MENA countries comprise a complex mosaic of populations and policies. Thus, each country requires a tailored approach to improving HIV surveillance and prevention efforts. Iran, for example, hosts the highest number of cases in the region (Figure) and is battling an epidemic driven primarily by injection drug use.^1,3,4^ It has implemented a set of inconsistent policies that has had both progressive and regressive impacts on the public health community’s efforts to control HIV transmission. For example, while Iran has served as a model for other countries in its successful implementation of harm-reduction policies and deployment of peer education programs and needle exchange centers,^5^ national laws have also been passed that are severely punitive against persons who inject drugs (PWIDs) and men who have sex with men (MSM).^6^ Saudi Arabia, in contrast to Iran, has among the lowest registered HIV cases in the world and leads the Arab Strategic Framework for the Response to HIV and AIDS,^7^ but pursues policies that confine and deport any incident cases among its sizeable guest worker population.^8,9^ Persons from the large guest working population who acquire HIV in the more tolerant United Arab Emirates still face the same expulsion policy. Conflicting policies and practices are not unique to specific countries, as competing priorities can yield contradictory approaches to societal problems, particularly in a region where traditional and modernizing influences create divergent sentiments.

**Figure:**
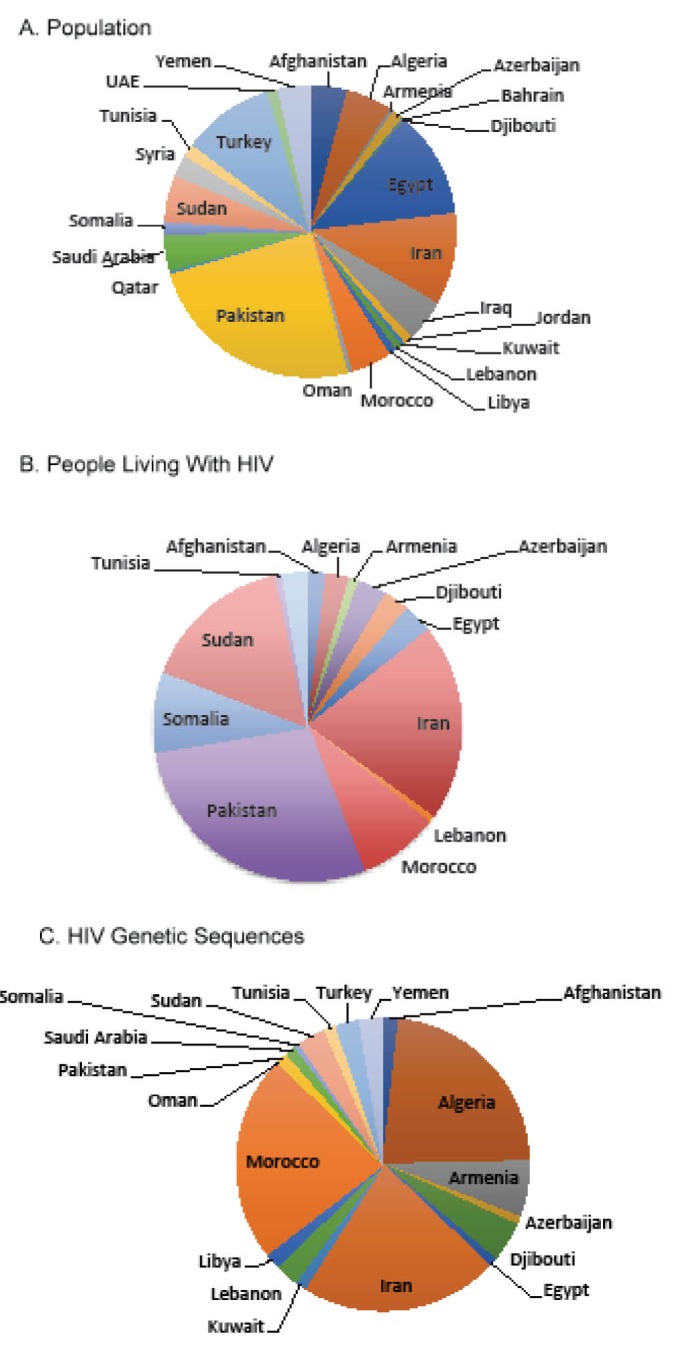
Distribution of Population, People Living With HIV/AIDS (B) and Published HIV-1 Sequences From Countries in the MENA Region. The total population (World Bank) (A), people living with HIV/AIDS (UNAIDS) (B), and number of sequences publicly available (GenBank) (C) in 2015 for the 25 countries in the MENA region. No HIV burden estimates (B) were available in 2015 for Bahrain, Iraq, Jordan, Kuwait, Libya, Oman, Qatar, Saudi Arabia, Syria, Turkey, and the UAE. No HIV sequences were publicly available for Bahrain, Iraq, Jordan, Oman, Qatar, Syria, and the UAE. Abbreviations: MENA, Middle East and North Africa; UAE, United Arab Emirates .

Advocates for enhanced HIV prevention, care, and treatment services in the MENA region face a conundrum: they require reliable data in order to persuade governments to pursue rational and consistent HIV prevention policies and invest more resources for HIV surveillance. How, then, can national policy makers in the MENA region be convinced that HIV is a public health threat that demands increased vigilance without verifiable evidence to justify such investments? Karamouzian et al suggest some initial steps toward tackling this problem, including the amplification of outreach efforts to regional refugees, MENA research training programs and cooperative initiatives to combat HIV-related stigma.^2^ Although these measures are necessary for a comprehensive strategy to rein in a worsening regional epidemic, they do not address the primary challenge of igniting stronger political will among regional health and finance leaders. In this commentary, we seek to enhance the excellent points presented by Karamouzian et al by suggesting additional ideas on how to create conditions that will promote national and international investments into enhanced data capture and validation systems that will ultimately lead to better control of HIV spread in the MENA region.

## Leverage Existing Data Systems and Public Health Infrastructures

New technologies, processes and protocols need not be invented *de novo* for HIV surveillance. There is already a network of extant surveillance, clinical and research data repositories, along with information-sharing agreements across the MENA region. There are several models from international governing bodies, national registries and not-for-profit organizations. A key example is the World Health Organization (WHO) Global Influenza Program that provides virologic and epidemiologic surveillance data on circulating strains and transmission patterns to member nations.^10^ Regional laboratories and sentinel sites that feed into this network have formed the basis for additional networks and collaborations that include the International Severe Acute Respiratory and Emerging Infection Consortium (ISARIC) and the Eastern Mediterranean Acute Respiratory Infection Surveillance Network (EMARIS).^11,12^ Since the emergence of the Middle East respiratory syndrome coronavirus (MERS-CoV) in 2012 on the Arabian Peninsula,^13^ these networks have been exploited for surveillance and monitoring of this and other novel respiratory pathogens. Laboratory infrastructures, database architectures, agreement frameworks, and operational logistics that have already been worked out for severe acute respiratory illnesses are models for how to handle regional data collection for HIV and other infectious diseases. Varying conditions have different epidemiologic features, but all require a network of laboratories, clinical centers and tracking systems under a coherent regional network plan.

Other examples outside the MENA region have shown that laying a broad foundation for disease surveillance can pay dividends for the control of epidemics of disparate nature. Amid the 2014 West African Ebola outbreak, the situation became frightening when this lethal filovirus appeared in Lagos, Nigeria, Africa’s most populous city with a 2016 population estimated by the National Population Commission of Nigeria to be 21 million persons. In spite of the high population density and unexpected emergence of Ebola in West Africa, Nigeria was able to halt its outbreak to 20 cases and eight deaths, far short of the tens-of-thousands of infections in the hardest hit countries of Guinea, Sierra Leone and Liberia.^14^ There were a number of reasons for Nigeria’s success. Paramount among them was the government’s ability to quickly turn its polio eradication program infrastructure to the task of containing Ebola transmission.^15^ If such a quick pivot can be made in the flux of a dynamic and widespread regional outbreak, then the same model could be adapted to the setting of the more slowly evolving and concentrated epidemic of HIV in the MENA region.

## Intensify the Follow-up, Care and Data Collection Efforts for Known HIV Cases

Despite a paucity of HIV surveillance data, Ministries of Health in the MENA region have the capability to track known HIV cases through their typically centralized care and treatment services.^4^ Still, these same countries collectively have the lowest antiretroviral coverage rates in the world, with an average of 17% on treatment, partially explaining the region’s 20-fold increase in AIDS-related deaths in the past 10 years.^1^ Although a centralized health system may have an effect of facilitating stigmatization, public health workers can also capitalize on this feature to capture more robust data and improve outcomes for their HIV infected patients. Although intensified surveillance and massively expanded HIV testing will be a key aspect to controlling the epidemic, a failure to capture key data on risk behaviors, treatment adherence, drug resistance and host and virus genetic sequences from known populations is a missed opportunity. Such data can inform policies to lower HIV incidence and mortality by helping target resources to where the largest impact could be expected, eg, MSM and sex workers (female, male, or transgender).

The MENA region is comprised of countries with a wide range of financial resources and technical capacity. Yet most of the data, particularly viral sequence diversity, comes from three middle-income countries: Algeria, Morocco, and Iran (Figure).^16^ Recently, several MENA countries have undertaken initiatives that put their genomic technologic prowess on full display. The Qatar Genome Program, for example, aspires to generate personalized genomic data for its entire population as part of a national precision medicine initiative.^17^ In an initial demonstration project, the program sequenced 1000 human genomes and uncovered more than 20 million polymorphisms to analyze. Yet, in the face of this titanic effort to sequence human genomes, not a single viral sequence from the approximately 100 documented HIV cases in Qatar has ever been entered into GenBank, as of April 2017.^18^ This incongruity argues that the tools and funding are available, but the political will is lacking for collecting basic virologic information on recognized HIV cases. The key will be in convincing policy-makers in the region that obtaining key data on subtype diversity and antiretroviral resistance mutations is straightforward and could guide the epidemiologic and clinical knowledge relevant for both HIV prevention and clinical care for persons living with HIV in the region.

## Engaging Key Populations

Throughout the history of the HIV/AIDS pandemic, key populations—including PWIDs, MSM, transgender populations, and female sex workers—have been subject to discrimination, stigmatization and criminalization. These policies drive away stigmatized subgroups and make it harder to engage them in the vital therapeutic and prevention alliances that enable implementation of successful health programs. Just as some health ministries outside the MENA region have adopted best practices from countries like Iran and Morocco,^5^ the MENA region could also learn lessons from countries with similar cultural conditions on how to best engage and reduce high-risk behaviors among key populations within their borders. One notable example comes from Nigeria, where homosexuality remains criminalized,^19^ yet also has well-established programs that collect data from and provides services to MSM populations.^20^ Given the cultural climate and standing laws in Nigeria, it is not surprising that this group has been difficult to contact, trace and recruit into research and treatment cohorts. Hence, researchers and public health practitioners have recruited key community leaders to lead the outreach efforts to members of their communities. It is likely that, in MENA countries, high-risk social and sexual networks are also best accessed and followed with the help of community leaders and/or peers already integrated into these networks; and that these efforts can be best operationalized with respondent-driven sampling, snowball sampling strategies or post-testing counseling of newly diagnosed persons.

## Conclusion

MENA investigators have shown that both key HIV-uninfected, vulnerable populations and HIV-infected persons can be studied with modern field and clinical methods. Prospective follow-up is feasible, molecular phylogeny is obtainable and interpretable for understanding transmission dynamics and surveillance can guide improved policies.^21-26^ History teaches time and time again that epidemics ignored are epidemics unleashed. A dearth of relevant data will simply misrepresent the true epidemic profile, misleading public health and clinical practitioners and policy-makers. Europe and North America, with decades of HIV successes and failures, can be allies, given their imperative to support global health for both humanitarian and self-serving interests.^27^

It has become clear that no region, no culture, and no population is immune to the emergence and expansion of an HIV epidemic. Despite bold claims in the past that HIV/AIDS is not a problem for predominantly Muslim nations, all indicators have pointed to rising HIV incidence and mortality, both in absolute numbers and in relation to the rest of the world. In the current commentary, we have elaborated on the argument made by Karamouzian et al that a key measure for preventing a concentrated epidemic from becoming a disseminated one is to intensively and consistently collect population-based data.^2^ Although recognition and characterization of a problem is the first step toward its resolution, it is meaningless if that knowledge does not translate into action. Action starts with active surveillance efforts and meaningful investments into regional networks and national programs, such as those that have been outlined in the course of this commentary. Efficiencies can evolve from repurposing existing tools, using translatable data-collection platforms and partnerships and adopting best surveillance practices. The story of HIV can be traced along a convoluted path of both maladaptive decisions and farsighted strategies. MENA countries have the opportunity now to move from the former to the latter, turning the tide of a potentially worsening public health crisis.

## Acknowledgements

Supported in part (Dr. Vermund) by NIH/NIMH grant P30 MH062294 (Center for Interdisciplinary Research on AIDS).

## Ethical issues

Not applicable.

## Competing interests

The opinions expressed herein are those of the authors and should not be construed as official or representing the views of the US Department of Defense or the Department of the Army.

## Authors’ contributions

KM and SHV conceptualized the paper; KM wrote the initial manuscript draft; Both authors revised and contributed to subsequent drafts of the manuscript; Both authors approved the final draft for submission.

## Authors’ affiliations

^1^U.S. Military HIV Research Program, Walter Reed Army Institute of Research, Silver Spring, MD, USA. ^2^Henry M. Jackson Foundation, Bethesda, MD, USA. ^3^Yale School of Public Health, Yale University, New Haven, CT, USA.
